# Evidence for treatment of muscular vein thrombosis in orthopaedic patients

**DOI:** 10.1007/s10195-013-0241-3

**Published:** 2013-05-07

**Authors:** Ioannis Pengas, William Nash, Natasha Reed, Sunil Kumar

**Affiliations:** 1 Princess Alexandra Hospital, Harlow, Essex, UK; 2 59 Westpoint Apts, Clarendon Rd, London, N8 0DB UK; 3 Queens Hospital, Romford, Essex UK

**Keywords:** Muscular vein thrombosis, Orthopaedic, Anticoagulation treatment, Isolated gastrocnemius or soleus calf vein thrombosis

## Abstract

**Background:**

Does below-knee symptomatic muscular (gastrocnemius or soleus) vein thrombosis (MVT) warrant investigation and treatment in post-operative orthopaedic patients? We performed a literature search and evaluated the evidence looking for guidance regarding this question.

**Materials and methods:**

We performed a literature search with the use of PubMed, Medline and Google Scholar from 1950 to September 2011. Search terms included “muscular vein thrombosis” (MVT) and “isolated gastrocnemius or soleus vein thrombosis” (IGSVT). We reviewed the eight level II studies relevant to our search, only one of which was in a specific orthopaedic population.

**Results:**

Studies looking at the rates of progression of isolated MVT have shown conflicting results. There is also a lack of consensus between studies that compare progression amongst groups with or without anticoagulant treatment. The majority of the studies do not distinguish between medical, surgical or orthopaedic patients.

**Conclusions:**

We cannot confidently recommend commencement of anticoagulation treatment upon identification of MVT in post-operative orthopaedic patients. We can only suggest that, once MVT is diagnosed, the patient should undergo serial ultrasound scan (USS) duplex scans, and if propagation is identified, then treatment may be deemed beneficial.

Level of evidence: III (review of non-randomized controlled cohort/follow-up studies).

## Introduction

Does below-knee symptomatic muscular vein thrombosis (MVT) warrant investigation and treatment in post-operative orthopaedic patients?

MVT and isolated gastrocnemius or soleus vein thrombosis (IGSVT) are often interchangeable terms used to describe thrombosis in the superficial veins of the soleus and gastrocnemius muscles of the calf. There is little agreement in the literature and clinical practice as to whether MVT should be classed as deep or superficial vein disease and thus whether the treatment falls into the current guidelines for deep vein thrombosis (DVT) or not. We noted that in some centres, when DVT is suspected, the below-knee muscular veins are routinely imaged during Doppler ultrasound, whilst in others they are not. This discrepancy has significant consequences for the patient, since current guidelines derived from the Cochrane Collaboration [1] recommend treatment of DVT with immediate subcutaneous low-molecular-weight heparin and then oral anticoagulation with warfarin for 3 months unless contraindicated. Therefore, orthopaedic patients are facing a lottery, where in some centres they are investigated and treated for MVT whilst in others they are not. There are currently no national guidelines as to whether these veins should be imaged routinely and which treatment patients should receive if any.

Thrombotic disease is a very real problem for orthopaedic patients; the rate of DVT without prophylaxis may be as high as 45–51 % [2]. An American study by White et al. [3] of primary hip (19,586) and knee (24,059) arthroplasties, of which 80–90 % received chemical prophylaxis, found that the incidence of DVT or pulmonary embolus (PE) within 3 months was 556 (2.8 %) and 508 (2.1 %), respectively. The figure quoted by the British Orthopaedic Association (BOA) on its recommended standardised consent form for total hip arthroplasty is a 2.5 % risk of DVT and <1 % for PE [4]. Warwick et al. [5] found that the incidence of fatal PE in a group of 1,162 total hip arthroplasties not receiving chemical prophylaxis was 0.34 %, whilst the rate of DVT was 1.89 %. These patients received antithrombotic stockings, and early mobilisation was encouraged. The long-term repercussions of DVT include post-thrombotic limb syndrome, chronic venous insufficiency, skin changes, pain, ulcers [6] and emboli including PE and possibly even death [7]. The Italian inter-society consensus statement on antithrombotic prophylaxis states that the incidence of venous thromboembolism (VTE) in Europe is approximately 770,000 [8].

**Fig. 1:**
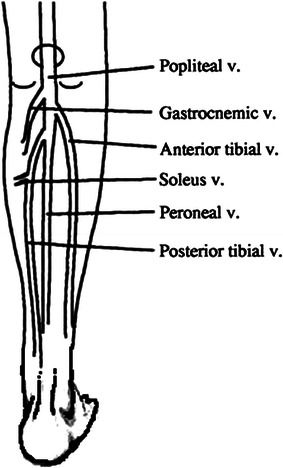
Schematic of distal calf veins (deep and muscular)

Distal or below-knee or calf vein thrombosis (DVT) refers to the anterior/posterior tibial, the peroneal veins, i.e. those that correspond to arterial structures. There are also the muscular calf (soleus or gastrocnemius) veins, as represented in Fig. 1. Kearon et al. [9] stated that, although the diagnosis of above-knee DVT is routinely performed by Doppler ultrasound scan (USS) with confidence (97 % sensitivity, 98 % specificity), the same cannot be said for below-the-knee diagnostic powers of this modality (50–75 % sensitivity, 90–95 % specificity). This limited performance of below-knee venous examination may explain why many centres investigate above-knee venous systems only.

## Materials and methods

We performed a literature search with the use of PubMed, Medline and Google Scholar from 1950 to September 2011. Search terms included “muscular vein thrombosis” (MVT) and “isolated gastrocnemius or soleus vein thrombosis” (IGSVT). All potentially relevant articles were retrieved and reviewed. Reference lists of the selected articles were reviewed, and pertinent publications were also included. Eight level II studies were found to have been conducted on MVT, one of which on orthopaedic patients alone.

In our review of the literature, we sought evidence for and against the treatment of MVT as an entity along with evidence for and against treatment in orthopaedic patients.

For the purpose of this paper, MVT is defined as combined thrombosis of gastrocnemius or soleus calf veins whereas isolated MVT refers to an isolated thrombosis of gastrocnemius or soleus calf veins, often referred to as isolated IGSVT.

## Results

### Muscular vein thrombosis

Gillet et al. [10] showed in a prospective level II study of 128 outpatients with isolated MVT that 29 cases developed further venous thromboembolic disease in a 36-month study period. Thrombus in above-knee deep veins occurred in 23 patients and pulmonary emboli in 6 patients. This paper does not distinguish between surgical and medical patients or their conditions.

MacDonald [11] studied the progress of 135 MVTs across all specialties in a prospective case series and found that the rate of propagation of MVT to the level of the popliteal vein was 3 %. All patients in whom this was the case had been diagnosed with cancer. Propagation in 90 % occurred within the first 2 weeks after diagnosis. None of these MVTs developed to involve a deep vein of the thigh or cause PE. Labropoulos et al. [12] concluded that the risk of propagation of soleus and gastrocnemius vein thrombosis to above-knee DVT was similar to that of posterior tibial and peroneal veins in their study of 48 patients, suggesting that MVT is perhaps of similar significance to other below-knee DVTs.

A prospective study of 84 consecutive patients with isolated MVT by Schwarz et al. [13] compared two cohorts of patients, one receiving treatment and the other not. Patients were monitored for thrombosis propagation with Doppler imaging, and it was found that in those treated for 10 days with heparin the rate of propagation was 25 % less than in those who were not (95 % confidence interval, 11.5–43.4 %). They had no cases of major haemorrhage in the group treated with anticoagulation. This study however did not distinguish between medical, surgical or orthopaedic patients. Sales et al. [14] in 2010 found no difference in 141 patients in the rate of progression of thrombus in isolated muscular calf vessels when comparing treatment with no treatment. Again this was in a pooled group of hospitalised patients. Galanuad et al. [15] compared 3-month outcomes of 222 deep vein thromboses and 390 muscular vein thromboses and found no difference in death or recurrence of VTE after treatment.

More recently, in 2009, Lautz et al. [16] retrospectively analysed 38,426 venous duplex scans at one institution over a 5-year period for patients with isolated IGSVT. They measured the rate of propagation amongst those patients who subsequently went on to have a repeat scan showing deep vein below-knee VTE, above-knee VTE or PE. This study showed an incidence of VTE of 18.7 % (DVT 16.3 %, PE 3.9 %, both 1.5 %). The authors found a significantly higher incidence of VTE in those who were not treated with therapeutic anticoagulation (no treatment, 30 %; prophylactic treatment, 27 %) when compared with those who were (12 %). The authors were unable to show the same association for PE. The analysis was carried out on 406 patients who were initially diagnosed with IGSVT and who then returned for subsequent studies. There were a further 296 patients who were diagnosed with IGSVT who were excluded from the analysis due to lack of follow-up (42 % overall). The retrospective nature of this study may well skew the incidence of propagation, as a huge proportion of patients were never followed up (presumably as they never subsequently developed a symptomatic VTE). Inclusion of these patients lost to follow-up could also significantly alter the incidence of VTE in the three different treatment groups if those lost to follow-up were less likely to have received treatment for their IGSVT. This study included all patients presenting for venous duplex scan.

### Muscular vein thrombosis in orthopaedic patients

Wang et al. [17] investigated a group of 359 consecutive patients in Taiwan who underwent total knee arthroplasty. All post-operative patients underwent imaging, and 175 patients (49 %) were found to have radiological evidence of DVT, 38 (22 %) of which were isolated MVT. They found that 16 (42 %) of the MVTs produced clinical symptoms but only 1 patient went on to develop propagation to above-knee DVT and none developed PE. Use of prophylaxis in these patients made no difference to the rate of late DVT, propagation or PE.

### MVT and DVT treatment in orthopaedic patients

Long-term consequences of below-knee DVT were studied by Masuda et al. [18] in a cohort study of 49 patients. Approximately half the group had received anticoagulation therapy for their thrombosis, whilst others had not. The incidence of propagation for the whole group was 4 % (two patients), and neither of these patients received thromboprophylaxis. They found that late complications of DVT, i.e. above-knee propagation and post-thrombotic syndrome, are low in all cases of isolated calf vein thrombosis at 3 years, and there were no cases of clinical PE. They agreed that, if propagation was to occur, it did so before 14 days.

Oishi et al. [19] studied a cohort of 273 consecutive orthopaedic patients who underwent total hip or knee arthroplasty and who received mechanical DVT prophylaxis with pneumatic compression stockings. Doppler imaging was used to detect, and subsequently follow up, progression of thrombus. Below-knee vein thrombosis developed in 41 patients (15 %), and all of these were asymptomatic. At days 7 and 14 post-operatively, they found that only seven patients (2.5 %) went on to develop above-knee thrombus, with all but one of these occurring within 14 days. While this study included all below-knee DVTs, it did not distinguish MVT separately. This evidence demonstrates that, while there is potential for thrombus propagation of below-knee DVTs, this usually occurs within the first 14 days.

A summary of these studies and their findings can be seen in Table 1.

**Table 1:** Summary of reviewed articles

Author	*n*	Study type	Population	Investigating	Outcome	Recommendations	Drawbacks	Overall for or against treating as per DVT
Gillet et al. [9]	128	Prospective observational study	Mixed outpatients	Rate of propagation in patients with therapeutic anticoagulation	18.8 % recurrence of VTE (including MVT) by 36 months; No recurrence occurred whilst on treatment	Need for standardisation of treatment	Asymptomatic MVT patients excluded	For
MacDonald et al. [10]	135	Prospective observational study	Mixed patients	Rate of propagation in patients without treatment	3 % rate of propagation to popliteal veins; No propagation to thigh veins or cases of PE; 90 % of propagation occurred within 2 weeks	Follow-up imaging beyond 2 weeks may not be necessary	Single-centre study	Against
Labropoulos et al. [11]	48	Prospective observational study	Mixed patients	Rate of propagation	When comparing DVT with MVT, the rate of propagation was similar	None	Small numbers	For
Schwarz et al. [12]	84	Randomized prospective	Mixed patients	Treatment vs. no treatment monitored for propagation	Rate of propagation 25 % less in those treated with 10 days of heparin	No benefit from compression stockings and LMWH vs. compression therapy alone in MVT patients	Small numbers; Largely ambulatory patient group (89 % outpatients)	Against
Sales et al. [13]	141	Non-randomised retrospective	Mixed inpatients	Treatment vs. no treatment monitored for propagation	No difference in rate of propagation when comparing treatment with no treatment	No difference in thrombus progression when comparing treatment with anticoagulation; Patients with other risk factors were more likely to show progression (ESRD, stroke)	Non-randomised; Ignored those patient who had isolated MVT but did not return for a subsequent scan	For
Lautz et al. [15]	406	Non-randomised retrospective	Mixed patients	Rate of propagation	10.4 % incidence of MVT; 19 % developed subsequent VTE; Significantly reduced by therapeutic (but not prophylactic) treatment	Therapeutic anticoagulation for MVT	Ignored the large proportion of patients with MVT who did not return for a subsequent scan (42 %)	For
Galanaud et al. [14]	390	Non-randomised retrospective	Mixed patients	Presenting symptoms; Rate of propagation	No difference in rate of propagation when comparing MVT with DVT	Consider DVT and MVT a homogeneous entity	Heterogeneous population from several centres; non-randomisation resulted in MVT being treated on fewer occasions than DVT (*P* = 0.003)	For
Wang et al. [16]	359	Non-randomized prospective	TKR patients	Incidence in orthopaedic patients; Rate of propagation	Rate of propagation similar when comparing DVT with MVT; Treatment made no difference to rates of DVT, propagation or PE	MVT should be considered comparable to DVT and treated as such	Stated as patient randomised to treatment, but not truly randomised; Conclusions drawn from differences in treatment groups are therefore flawed	For

*ESRD* end-stage renal disease, *LMWH* low molecular weight heparin, *TKR* total knee replacement

## Discussion

There are potential complications for orthopaedic patients taking anticoagulation treatment, including haemorrhage [locally, from the gastrointestinal (GI) tract or intracranially], infection, wound breakdown, warfarin-induced skin necrosis [20] and prolonged hospital stay. A study by Saleh et al. [21] found that haematoma formation and persistent post-operative drainage increase the risk of superficial surgical-site infection, and this in turn is strongly related to deep wound infection. Patel et al. [22] have found that persistent post-operative drainage is associated with the use of low-molecular-weight heparin. An American study by Novicoff et al. [23] published in 2008 retrospectively examined over 1,000 orthopaedic cases undergoing total hip or knee arthroplasty over 3 years. According to local protocol introduced in 2005, all patients received warfarin starting on the day of surgery and low-molecular-weight heparin if they were at high risk of thrombotic disease to continue for 4–6 weeks after surgery. The implementation of this regimen saw a statistically significant increase in the rate of complications and length of hospital stay. Bleeding complications increased from 1.4 % in 2004 to 9.6 % in 2006 (*P* < 0.0001). The rate of venous thromboembolism did not change in this period. The studies that look at complications of anticoagulation for orthopaedic patients focus largely on prophylaxis rather than treatment regimens. We speculate that the range and rate of complications may differ since the treatment dose is higher and for a longer period.

The cost of primary DVT treatment in the UK is calculated to be £721 [24], including diagnosis, anticoagulation and follow-up. For lifetime treatment of post-thrombotic limb syndrome, this rises drastically to £3,866, making treatment of DVT worthwhile. However, the cost of treating just one episode of major haemorrhage is estimated to be over £10,000 [25]. It is not clear whether the same long-term consequences apply to MVT when considered as a separate entity from DVT. Since there is no uniform classification of MVT, it is unclear whether much of the data on the risks versus benefit of DVT treatment apply to MVT or not.

The diagnosis and treatment of DVT for any patient is significant, with a minimum of 3 months anticoagulation, regular blood tests, subcutaneous heparin or warfarin with multiple visits to hospital or clinics. Anticoagulation treatment in DVT patients is also associated with a major haemorrhagic risk (0.6–1.2 %) and fatal bleeding (0.1–0.4 %) over the 3-month treatment period depending on treatment method [26]. In addition, such patients will be labelled high risk for further thrombotic events according to National Institute for Clinical Excellence (NICE) guidelines [27], meaning that for subsequent admissions they will always be given chemical prophylaxis. This would also be the case in other parts of the world where national guidelines or consensus statements exist regarding managing the risk of VTE in orthopaedic patients. Another example of this would be the Italian inter-society consensus statement on antithrombotic prophylaxis [8].

Attached to this diagnostic label is the disadvantage of being considered a higher risk when determining life and health insurance. Patients in the USA report having their monthly premiums doubled on finding they had a DVT [28]. Such a diagnosis will be permanently recorded in the patient’s medical notes and affect how they are treated on subsequent hospital admissions. To label MVT as DVT therefore has significant implications for the patient’s future.

In a review article by Righini et al. [29], prospective outcome studies for above-knee and complete (above and below knee) DVT investigations were scrutinised. This demonstrated that the pooled 3-month thromboembolic risk of the above knee and the combined (above and below knee) were similar, stating that detection of calf DVT may not reduce the 3-month thromboembolic risk but entails a significant risk of false-positive findings with subsequent unnecessary anticoagulation.

In summary, studies looking at the rates of progression of isolated MVT have shown conflicting results. There is also a lack of consensus between studies that compare progression amongst groups with or without anticoagulant treatment. The majority of the studies do not distinguish between medical, surgical or orthopaedic patients. When surgical, and in particular lower limb arthroplasty patients are considered separately, the results may well differ as the underlying risk factors are quite different. Therefore, the rate of propagation and long-term effects will also be affected by this method of patient selection. There are difficulties in drawing together the evidence that exists to analyse an overall risk, since the studies use very different variables such as patient group, timescale, interventions and investigations.

Considering the absence of national guidelines and level I evidence, the small number of studies and the well-documented potential complications of anticoagulation in the orthopaedic group of patients, MVT should not be routinely treated. We can only recommend surveillance of MVT with serial Doppler investigations for a period of no less than 2 weeks in order to identify those clots which propagate proximally and to only start anticoagulation treatment if propagation occurs.

Finally, we suggest that a high level of evidence trial, such as a randomized control trial, be conducted to ascertain whether diagnosis of MVT in an orthopaedic group of patients is significant, and if treatment of MVT in orthopaedic patients is indicated.
